# Unexpected Fluorescence Emission Behaviors of Tetraphenylethylene-Functionalized Polysiloxane and Highly Reversible Sensor for Nitrobenzene

**DOI:** 10.3390/polym13183046

**Published:** 2021-09-09

**Authors:** Lianfeng Wu, Qin Jiang, Haifeng Lu, Shengyu Feng

**Affiliations:** Key Laboratory of Special Functional Aggregated Materials, Ministry of Education, School of Chemistry and Chemical Engineering, Shandong University, Jinan 250100, China; wulianfeng126@126.com (L.W.); jiangqin199212@163.com (Q.J.)

**Keywords:** tetraphenylethylene, polydimethylsiloxane, aggregation-induced emission, fluorescent probe

## Abstract

Tetraphenylethylene (TPE), a typical luminogen with aggregation-induced emission (AIE) features, has been widely used to prepare AIE fluorescent materials. In this study, TPE-functionalized polydimethylsiloxane (n-TPE-AP-PDMS) was successfully synthesized by attaching TPE to polydimethylsiloxane via aza-Michael addition. The introduction of polydimethylsiloxane to TPE had no obvious effect on photophysical properties. Intriguingly, n-TPE-AP-PDMS exhibited two opposite fluorescence emission behaviors in different systems: aggregation-induced quenching (ACQ) behavior in a tetrahydrofuran/water mixture and typical AIE phenomenon in a tetrahydrofuran/hexane mixture. This unexpected transition from ACQ to AIE can be attributed to a twisted intramolecular charge-transfer effect and flexible aminopropyl polydimethylsiloxane. n-TPE-AP-PDMS was further used as a fluorescent probe to detect nitrobenzene and it showed high quenching efficiency. Moreover, the n-TPE-AP-PDMS film showed high reversibility so that the quenching efficiency remained constant after five cycles. This work can provide a deeper understanding of AIE behavior and guidance to develop a new AIE polymer for chemosensors with high performance.

## 1. Introduction

Fluorescent materials have attracted much attention due to their fundamental and indispensable roles in human society. The fluorescence emissions of traditional luminescent materials obviously decrease or even completely quench in aggregated state due to the intrinsic aggregation-caused quenching (ACQ) feature, greatly limiting their applications in high concentration or a solid state. Aggregation-induced emission (AIE), which is exactly opposite to the ACQ phenomenon, was first reported by Tang and co-workers in 2001 [1,2]. Restriction of the intramolecular rotation (RIR) mechanism is considered to be primarily responsible for the AIE phenomenon. Due to their excellent fluorescent properties, AIE luminogens have been extensively applied in various fields, including bioassay [3], membrane imaging [4], explosive detection [5], light-emitting diodes (OLEDs) [6] and liquid crystal materials [7].

Tetraphenylethylene (TPE) has a simple molecular structure but shows an obvious AIE feature. TPE can be facilely incorporated into traditional fluorescence molecules, converting ACQ molecules to AIE luminogens. Therefore, a large variety of new AIE luminogens based on the TPE unit have been successfully developed. Tang’s group [8] reported the synthesis and multilayer electroluminescence (EL) applications of triphenylamine-containing tetraphenylethylenes, which showed typical AIE phenomenon and high morphological stability. Wang and co-workers [9] reported an AIE-active TPE derivative, utilized as a highly sensitive fluorescent probe for lipid droplet imaging and tracking. Kang and co-workers [10] synthesized a near-infrared AIE luminogen by decorating the TPE core with an electron donor and electron acceptor, which possessed high specificity, good biocompatibility and excellent photostability.

Fluorescent polymers with flexible design of molecular structure and morphology as well as high thermal stability are ideal candidates to develop new AIE systems. TPE-based AIE polymers exhibit the advantages of a typical AIE effect and high thermal stability, which meet the essential requirements of practical applications, such as chemosensors and light-emitting diodes [11]. Wu and co-workers [12] reported a series of polyfluorene-containing TPE moieties in the main chain, which are used as excellent chemosensors and light-emitting materials. Li and co-workers [13] attached TPE units onto methylvinyldiethoxylsiloxane to synthesize a polysiloxane with TPE pendants, which possessed high thermal stability and morphological stability. The organic reaction to attach TPE moiety on the polymer skeletons becomes the key role to efficiently prepare AIE polymer. He and co-workers [14] reported a spontaneous aza-Michael addition polymerization for efficient preparation of poly(enamine)s, which could proceed with 100% atom efficiency under mild conditions without any external catalyst. Therefore, aza-Michael addition is an ideal strategy to prepare TPE-based fluorescent polymer.

In this work, TPE-functionalized polymers (n-TPE-AP-PDMS) were facilely synthesized by TPE derivative (n-TPE) and side-chain aminopropyl polydimethylsiloxane (AP-PDMS) via aza-Michael reaction. The TPE derivative (n-TPE) was dispersed uniformly in the side-chain of polydimethylsiloxane and the thermal stability was significantly enhanced. Moreover, the fluorescence behaviors of n-TPE-AP-PDMS in different mixtures (tetrahydrofuran/water and tetrahydrofuran/hexane) were systematically investigated. As TPE-pendant polymers had good film-forming ability, the subsequent film used as a fluorescent probe for detecting nitrobenzene was also studied. Investigation of the fluorescence-quenching behavior indicated that n-TPE-AP-PDMS film had detection sensitivity as well as good reversibility.

## 2. Experimental Section

### 2.1. Materials and Instrumentations

All reagents and solvents were purchased from commercial sources and used without further purification except for tetrahydrofuran, which was distilled over sodium and benzophenone before use. Side-chain aminopropyl polydimethylsiloxane with amino content of 2% (M_W_ = 39894 g∙mol^−1^, according to GPC (Shimadzu, Tokyo, Japan) analysis) was synthesized by ring opening polymerization.

^1^H (400 MHz) and ^13^C (101 MHz) NMR data were recorded on a Bruker Avance 400 MHz (Karlsruhe, Germany) spectrometer using chloroform-d or DMSO-d_6_ as solvent and tetramethylsilane (TMS) as internal standard. High-resolution mass spectra were obtained using positive mode on Agilent Technologies 6510Q-TOFLC-MS (Santa Clara, CA, USA). The UV–vis and fluorescence spectra were recorded on a TU-1901 double-beam UV–visible spectrophotometer (Peking, P.R. China) and a Hitachi F-7000 spectrophotometer (Tokyo, Japan), respectively. Absorption and emission measurements were conducted on samples in 1 cm × 1 cm quartz cuvettes. All experiments were performed at room temperature (~25 °C) unless otherwise specified. Fourier transform infrared spectra (FT-IR, Karlsruhe, Germany) were recorded on a Bruker TENSOR27 infrared spectrophotometer using the KBr pellet technique within the 4000–400 cm^−1^ region. Scanning electronic microscopy (SEM) image was obtained using Hitachi S-4800 (7 kV) (Tokyo, Japan). Thermal gravimetric analyses (TGA) were performed with a Mettler Toledo SDTA-854 TGA system (Zurich, Swiss) in nitrogen at a heating rate of 10 °C∙min^−1^. DSC measurement was studied using DSCQ 2000 of TA Instruments (TA Instruments, New Castle, DE, USA) with heating rate of 10 °C∙min^−1^ under nitrogen atmosphere.

### 2.2. Fluorescence Quenching Investigation

The n-TPE-AP-PDMS film was prepared by solvent volatilization of the n-TPE-AP-PDMS in THF solutions on a quartz substrate. The dried n-TPE-AP-PDMS film was then put on the top of a sealed bottle which was filled with saturated nitrobenzene vapor. The fluorescence quenching investigations of the n-TPE-AP-PDMS film to the vapor of nitrobenzene were carried out at room temperature.

### 2.3. Synthesis

#### 2.3.1. Synthesis of 1-(4-Hydroxyphenyl)-2,2-bis(4-dimethylaminophenyl)-1-phenylethene

Zinc dust (12.94 g, 198 mmol), 4-Hydroxybenzophenone (7.128 g, 36 mmol) and 4,4′-Bis(dimethylamino)benzophenone (8.04 g, 30 mmol) were placed into a 250 mL flask equipped with a reflux condenser. The mixture was evacuated under vacuum and filled with dry nitrogen. Dry THF (150 mL) was added and the mixture was cooled to −15 °C and TiCl_4_ (10.88 mL, 99 mmol) was slowly added. The mixture was warmed to room temperature, stirred for 0.5 h, and heated to reflux overnight. The reaction was quenched with a 10% aqueous solution of K_2_CO_3_ and a large amount of water was added to the solution until the solid turned gray. The mixture was extracted with dichloromethane three times and the organic layers were combined and washed with brine twice. After evaporating the solvent under reduced pressure, the crude product was further purified by column chromatography on silica gel by using a mixture of Hex/EA (15:1 *v*/*v*) as elute. The product 1 was obtained as a yellow solid (2.83 g). The yield was 22%. ^1^H NMR(400 MHz, DMSO), δ(ppm): 9.21 (s,1H), 7.24–6.90 (m,5H), 6.73 (ddd, *J* = 8.8, 6.3, 2.1, 6H), 6.61–6.38 (m, 6H), 2.86–2.79 (m, 12H). ^13^C NMR (101 MHz, DMSO), δ(ppm): 155.70, 145.62, 140.60, 137.28, 135.56, 132.45, 132.39, 132.34, 132.25, 132.13, 128.08, 125.46, 115.11, 111.73, 49.07. HRMS(ESI): Calcd for C_30_H_30_N_2_O: 435.2358 [M + H]^+^; found: 435.2448.

#### 2.3.2. Synthesis of 1-(4-Acryloxyphenyl)-2,2-bis(4-dimethylaminophenyl)-1-phenylethene (n-TPE)

To a 250 mL round-bottom flask, compound 1 (2.4 g, 5.52 mmol) and triethylamine (0.56 g, 5.52 mmol) were added and dissolved in 100 mL of dichloromethane. The mixture was stirred and cooled to 0 °C. A solution of acryloyl chloride (0.51 g, 5.6 mmol) in 10 mL dichloromethane was added dropwise to the mixture. The mixture was warmed to room temperature, stirred for 3 h. After evaporating the solvent under reduced pressure, the crude product was purified by column chromatography on silica gel by using a mixture of Hex/EA (15:1 *v*/*v*) as elute. The product (n-TPE) was obtained as a yellow solid (2.21 g, yield: 82%). ^1^H NMR(400 MHz, CDCl_3_), δ(ppm): 7.10–6.90 (m, 7H), 6.81 (d, *J* = 8.6, 6H),6.49 (dd, *J* = 17.3, 1.2, 1H), 6.40 (s, 4H),6.21 (dd, *J* = 17.3, 10.4, 1H), 5.90 (dd, *J* = 10.4, 1.2, 1H),2.84 (s, 12H). ^13^C NMR (101 MHz, CDCl_3_), δ(ppm): 164.43, 147.72, 145.69, 139.56, 137.04, 135.72, 134.25, 132.61, 132.41, 132.32, 132.26, 131.54, 127.5, 121.5, 120.56, 112.11, 111.7340.06. HRMS(ESI): Calcd for C_33_H_32_N_2_O_2_: 489.2464 [M + H]^+^; found: 489.2488.

#### 2.3.3. Synthesis of n-TPE-AP-PDMS

AP-PDMS and n-TPE (the molar ratio of amino of aminopropyl moiety and vinyl group of acryloxy moiety were 1:1) were dissolved in 20 mL dichloromethane and added into a 100 mL round-bottom flask equipped with a reflux condenser. The mixture was stirred and heated to reflux for 8 h. After cooling to room temperature, the solvent was removed under reduced pressure. The product was obtained as yellow viscous liquid. FT-IR (cm^−1^): 1886, 1260, 1020, 1097, 868, 798. ^1^H NMR(400 MHz, CDCl_3_), δ(ppm): 7.65–6.99(Ar*H*), 3.72 (–C*H*_2_–C=O), 3.51 (–N–C*H*_3_), 2.80 (–N*H*–C*H*_2_–CH_2_–C=O), 2.61 (–NH–CH_2_–C*H*_2_–C=O), 2.37 (–NH–C*H*_2_) 1.64 (–N*H*–CH_2_–C*H*_2_), 1.26 (–Si–CH_2_–C*H*_2_), 0.90 (–Si–C*H*_2_–CH_2_), 0.09 (–Si–C*H*_3_).

## 3. Results and Discussion

### 3.1. Synthesis and Characterizations

**Scheme 1:**
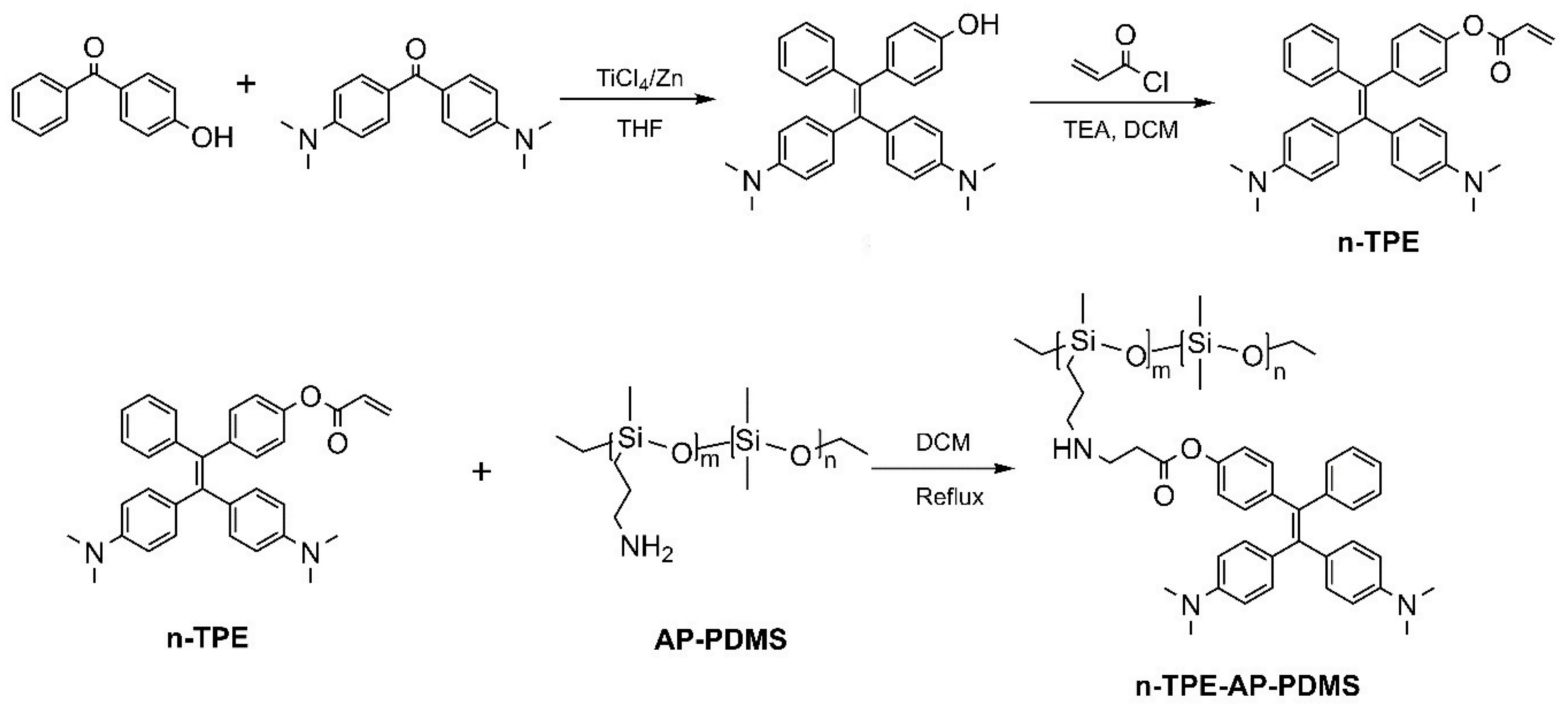
Synthetic procedure of n-TPE-AP-PDMS.

n-TPE was attached to AP-PDMS polymer chains via aza-Michael addition reaction and the synthetic process were shown in Scheme 1. FT-IR spectra of the n-TPE and n-TPE-AP-PDMS are presented in the Supporting Information (Figure S1). For n-TPE, a peak at 1886 cm^−1^, which was attributed to C=C in acryloxy moiety, was observed. The double bond (C=C) disappeared in n-TPE-AP-PDMS, suggesting the aza-Michael reaction was completed. The ^1^H NMR spectrum also confirmed the structure (Figure S2). The microphase separation of the obtained film was not observed by the scanning electronic microscopy (SEM) as shown in Figure S3, which meant the n-TPE was dispersed uniformly in side-chain aminopropyl polydimethylsiloxane without the formation of large aggregates.

### 3.2. Thermal Stability

**Figure 1:**
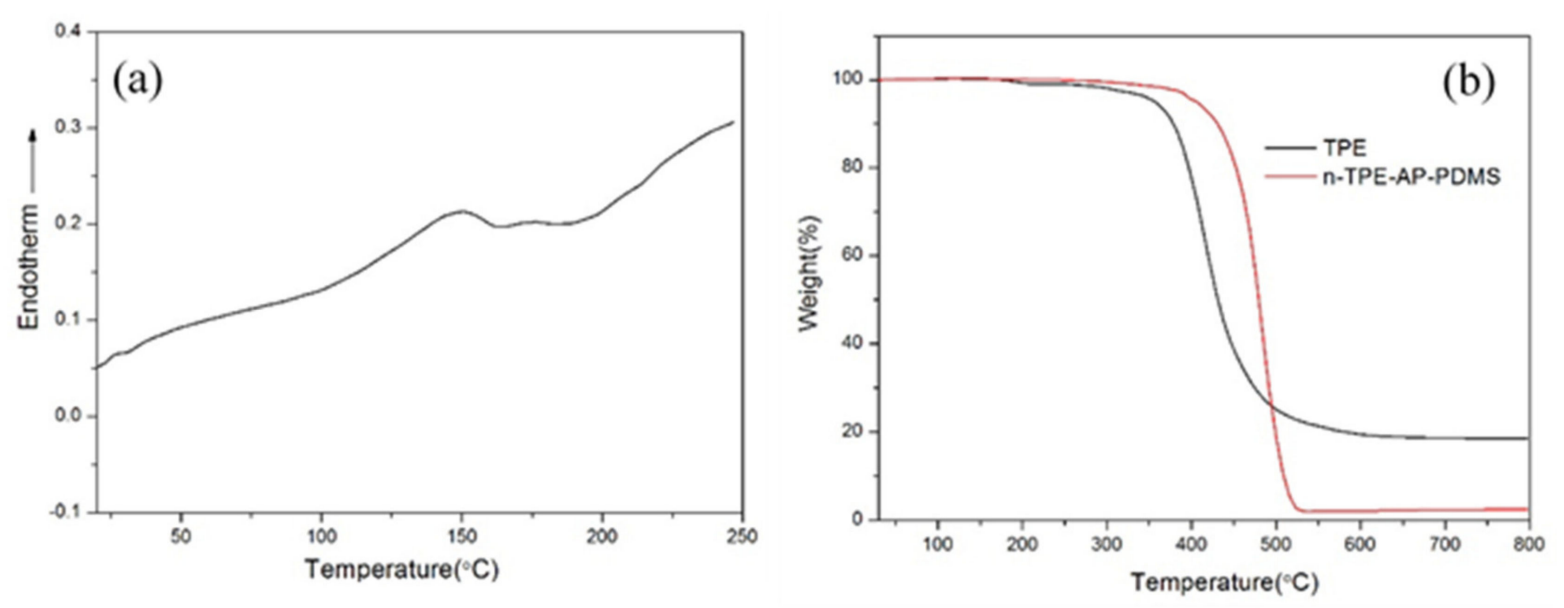
(**a**) DSC trace of n-TPE-AP-PDMS; (**b**) TGA trace of n-TPE-AP-PDMS and n-TPE recorded at a heating rate of 10 °C∙min^−1^ under nitrogen atmosphere.

The thermal properties of n-TPE-AP-PDMS were characterized by DSC and TGA under nitrogen atmosphere. As shown in Figure 1, the DSC traces showed the glass transition temperature (T_g_) of 142 °C. Moreover, neither exothermic peak nor endothermic peak was observed over the investigated temperature range (25–250 °C). These results indicate that n-TPE-AP-PDMS is a stable amorphous material. Meanwhile, the decomposition temperature of the n-TPE-AP-PDMS, which corresponds to a weight loss of 5% at a heating rate of 10 °C∙min^−1^ was measured to be 403 °C and the peak decomposition temperature was around 530 °C in Figure 1a. In comparison, the TGA analysis of the n-TPE was also carried out (Figure 1b). The thermal decomposition temperature of n-TPE was located at 357 °C, which was much lower than the result of the n-TPE-AP-PDMS. This indicates that the thermal stability of AIE luminogen is effectively enhanced by attaching to the AP-PDMS due to the high chemical-bond energy (Si–O–Si, 422.5 kJ∙mol^−1^). The high glass transition and thermal decomposition temperature suggest the good thermal stability of n-TPE-AP-PDMS.

### 3.3. Photophysical Properties

**Figure 2:**
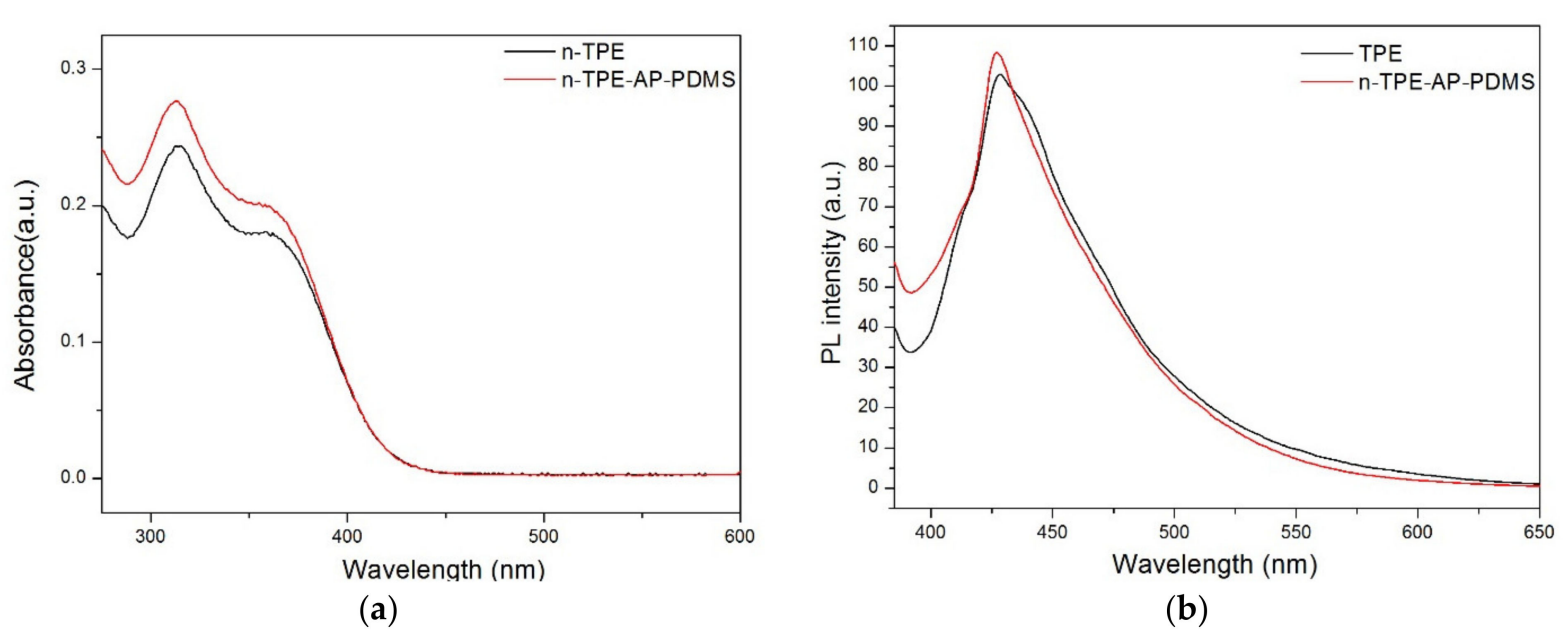
(**a**) The UV–vis absorption spectra of n-TPE and n-TPE-AP-PDMS in THF; (**b**) The photoluminescence (PL) spectra of n-TPE and n-TPE-AP-PDMS in THF; λex = 375 nm; concentration: 10 μM.

The absorption properties of n-TPE and n-TPE-AP-PDMS were first investigated in dilute THF solutions. In UV-vis absorption spectra (Figure 2a), the absorption maximum (λ_max_) of n-TPE and n-TPE-AP-PDMS were both centered at 362 nm, which is derived from π-π* transition of TPE moiety [15]. The photoluminescence (PL) spectra of n-TPE and n-TPE-AP-PDMS in the THF solution at the low concentration of 10 μM upon excitation at 375 nm in Figure 2b were almost the same. These results indicated that introducing polydimethylsiloxane into the TPE moiety had no effect on the photophysical properties of TPE moiety.

### 3.4. AIE Behavior

**Figure 3:**
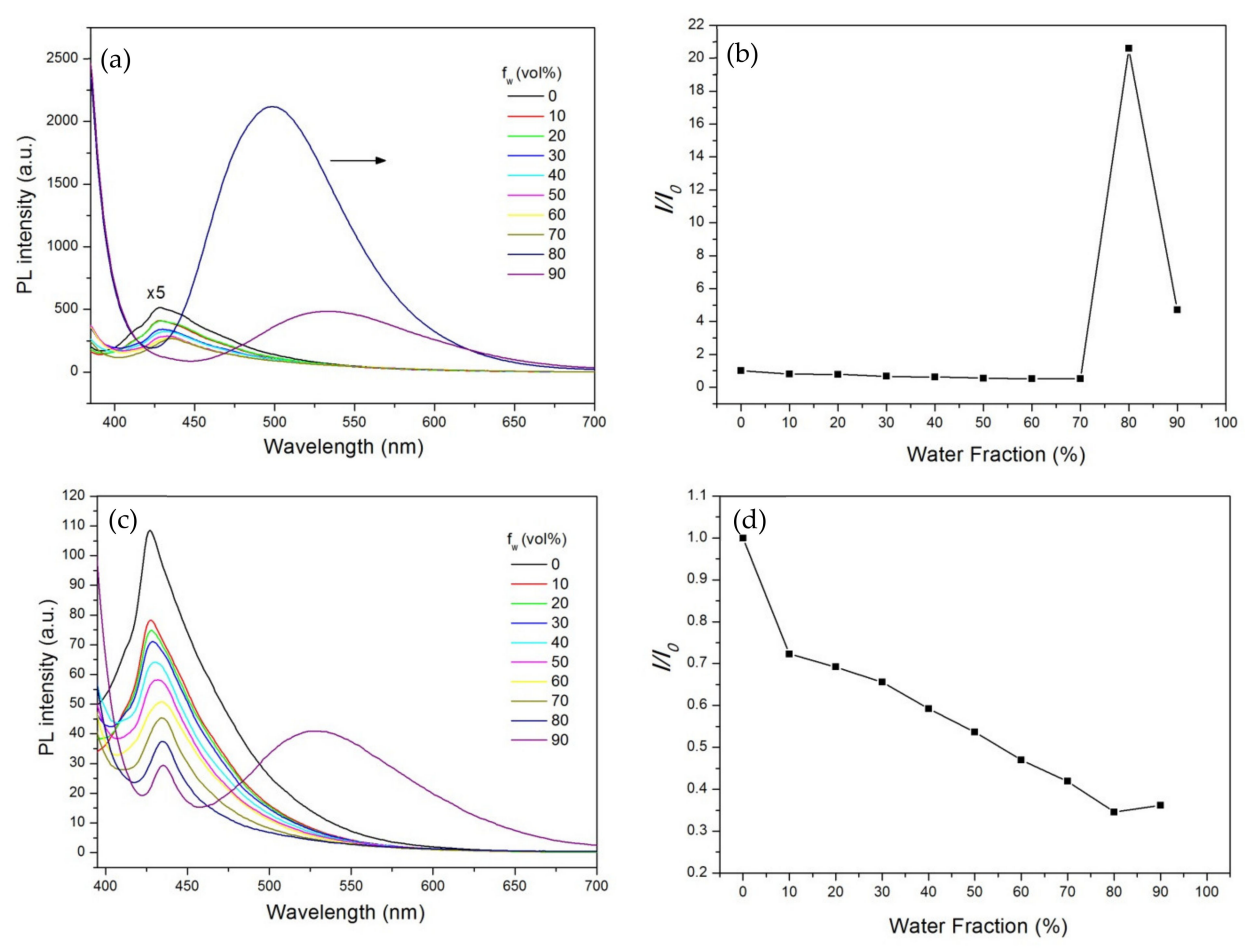
(**a**,**b**) PL spectra and changes in relative PL intensity versus different water fractions of n-TPE (The chosen wavelength for the calculation of “*I*/*I*_0_” is 430 nm for 0–70%, 498 nm for 80%, 532 nm for 90%); (**c**,**d**) n-TPE-AP-PDMS in THF/water mixtures. λex = 375 nm; concentration: 10 μM. (The chosen wavelength for the calculation of “*I*/*I*_0_” is 430 nm for 0–80%, 533 nm for 90%).

To identify the difference between the n-TPE and n-TPE-AP-PDMS in terms of AIE performance, we studied the fluorescence emission behaviors of n-TPE and n-TPE-AP-PDMS in THF/water mixtures at the concentration of 10 μM. With the addition of water (a poor solvent), the emission peak of n-TPE gradually decreased with a red-shift (Figure 3a). Specifically, more than 48% of its fluorescence intensity was quenched at the water fraction (f_w_) of 70%. Such emission quenching can be attributed to the enhancement of the twisted intramolecular charge-transfer (TICT) effect from the dimethylamino (an electron donor) group to the acryloxy (an electron acceptor) group of n-TPE owing to the increase in the solvent polarity with water addition. When the water fraction increased to 80%, the PL intensity of n-TPE was 20.59-fold higher than that in the pure THF solution. The enhanced emission could be attributed to the aggregate formation that activates the aggregation-induced emission channel. The weaker emission of n-TPE at f_w_ of 90% compared with that at f_w_ of 80% is probably due to the difference in aggregate morphology (Figure 3b).

Unexpectedly, the fluorescence emission variations of n-TPE-AP-PDMS in THF/water mixtures were completely different from n-TPE. As shown in Figure 3c,d, the fluorescence intensity of n-TPE-AP-PDMS showed a typical ACQ characteristic. With the increase in water fraction, the fluorescence intensity gradually decreased. When the water fraction was 90%, the emission intensity decreased to 34% of the original intensity. Since the results of UV–vis absorption and PL spectra suggested that introducing polydimethylsiloxane into the TPE had no effect to the photophysics of the TPE derivative (Figure 2a,b), we proposed the reason for this was the effect of the type of poor solvents on the n-TPE-AP-PDMS’s behavior in mixtures.

**Figure 4:**
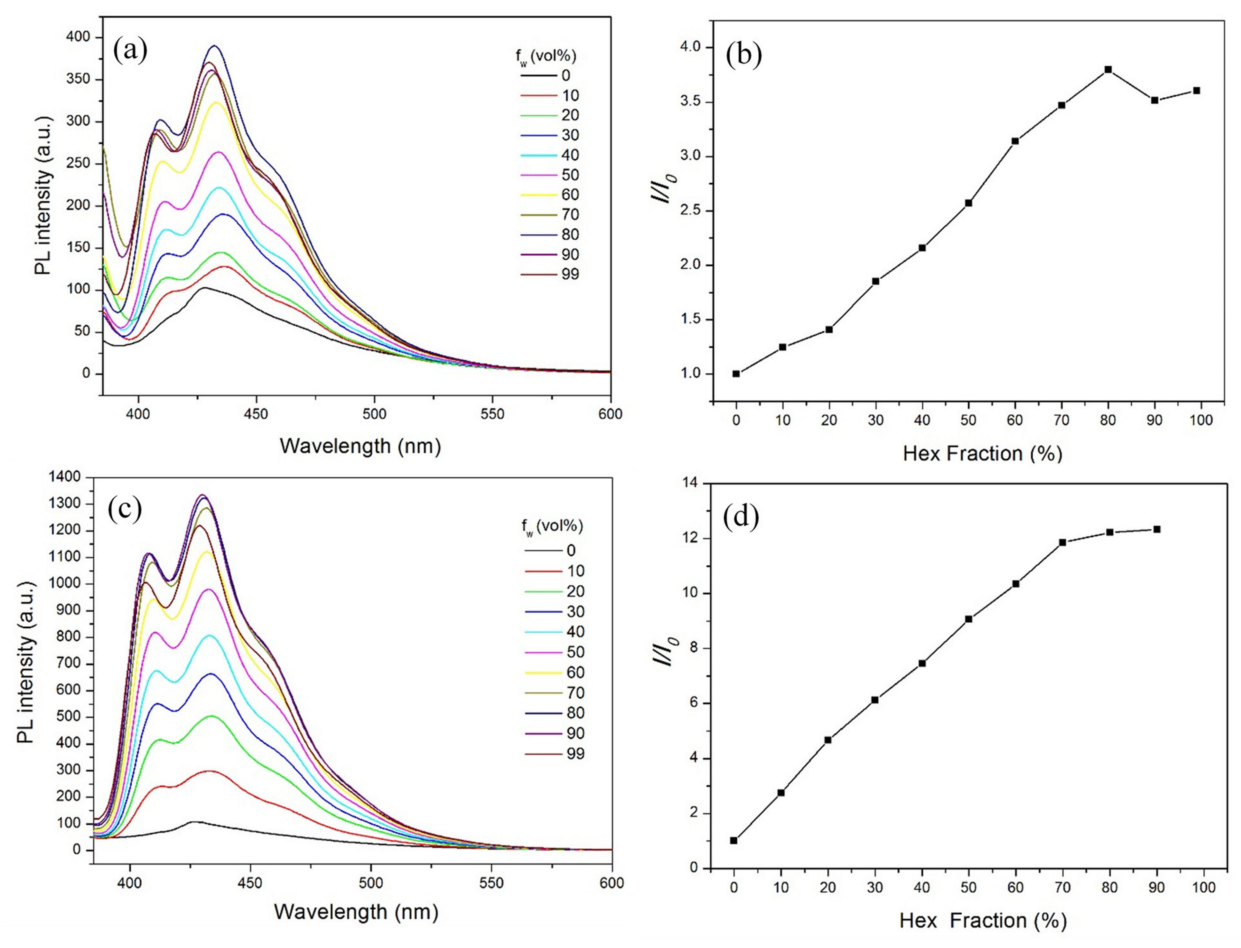
PL spectra and changes in relative PL intensity versus different water fractions of n-TPE (**a**,**b**) and n-TPE-AP-PDMS (**c**,**d**) in THF/n-hexane mixtures. λex = 375 nm; concentration: 10 μM. (The chosen wavelength for the calculation of “*I/I_0_*” is 435 nm).

Therefore, we chose n-hexane as the poor solvent instead of water to further study n-TPE and n-TPE-AP-PDMS’s fluorescence emission behaviors in THF/n-hexane mixtures and the results are shown in Figure 4. n-TPE still showed AIE behavior in THF/n-hexane mixtures with the highest value of the intensity ratio *I*/*I*_0_ (*I* is PL intensity in THF/n-hexane mixtures with different n-hexane fractions, *I_0_* is PL intensity in pure THF solution) of 3.80. For n-TPE-AP-PDMS, the addition of n-hexane into the THF solution induced the molecules to aggregate and gradually enhanced its PL intensities. The highest emission intensity was observed in 99% mixtures and its intensity was enhanced about 12.33-fold compared with that of its THF solution, demonstrating a typical AIE phenomenon (Figure 4). These results indicated that n-TPE-AP-PDMS showed two opposite fluorescent behaviors in different mixture systems: ACQ in THF/water mixtures and AIE in THF/hexane mixtures. The ACQ behavior can be attributed to THF/water mixtures. As the water fraction increased in THF/water mixtures, the twisted intramolecular charge-transfer (TICT) effect from the dimethylamino (electron donor) group to the acryloxy (electron acceptor) group of n-TPE was enhanced owing to the increase of the solvent polarity with water addition, leading to a significant decrease in the fluorescence emission. In addition, as n-TPE also exhibited AIE behaviors in THF/water mixtures, the polydimethylsiloxane obviously had an important contribution to the ACQ phenomenon. We inferred that the flexible aminopropyl polydimethylsiloxane, which is slightly soluble in water, can partly suppress the aggregation formation in THF/water mixtures. As shown in Figure S4, the PL intensity of n-TPE-AP-PDMS showed a slight change in solvents with different polarity. Therefore, the significant increase in PL emission in THF/hexane mixtures can be mainly attributed to the AIE behavior of n-TPE-AP-PDMS.

### 3.5. Explosive Detection

Nitrobenzene, as a nitroaromatic compound, is harmful to the environment and humans because of its explosive and carcinogenic nature [16,17,18,19]. Thus, the detection of nitrobenzene is highly desirable in both the social security and human health field. Several methods have been employed to detect nitrobenzene, including gas chromatography coupled with mass spectrometry [20], nuclear quadrupole resonance spectroscopy [21] and energy-dispersive X-ray diffraction [22]. Among them, the fluorescence probe is economic and provides easy visualization, which has become a hot topic in explosive detection [23,24,25,26]. The fluorescence response of n-TPE-AP-PDMS film in detecting nitrobenzene was investigated.

**Figure 5:**
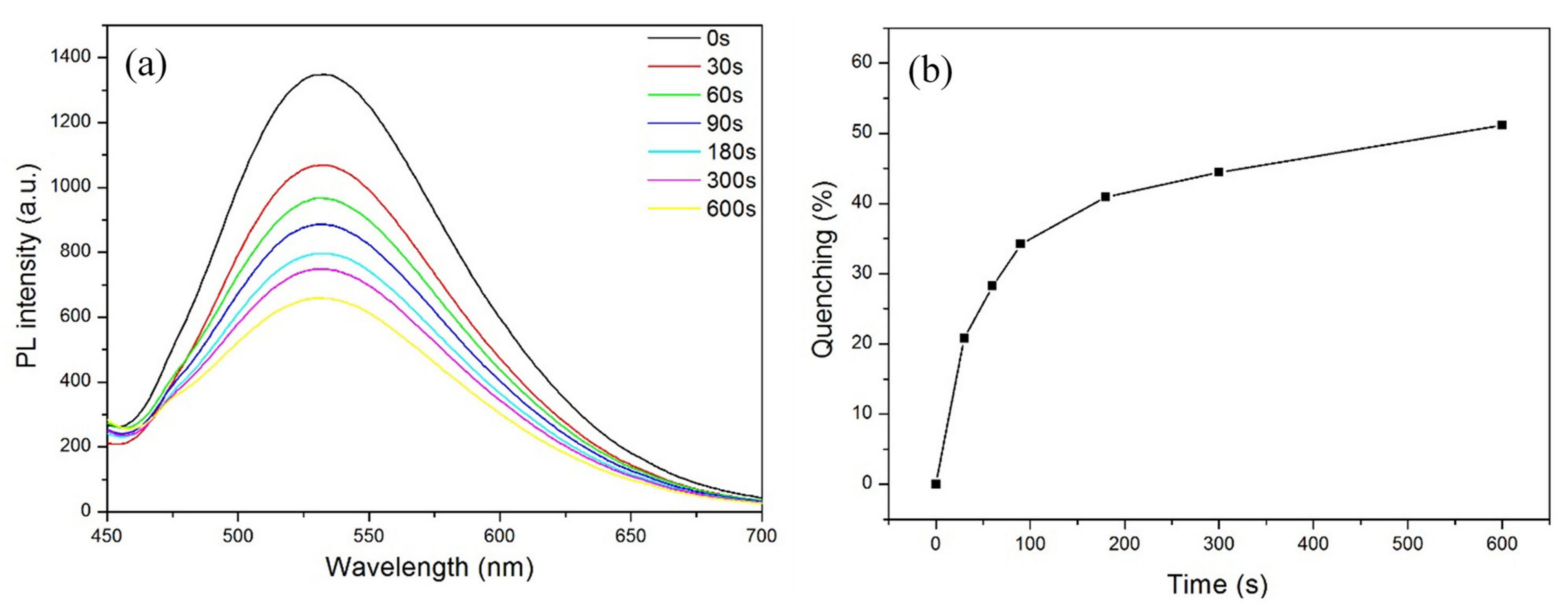
(**a**) Time-dependent PL intensity of n-TPE-AP-PDMS film in NB vapor; (**b**) fluorescence quenching curve in NB at different times.

The n-TPE-AP-PDMS film was prepared via facile solvent evaporation. Then, the obtained film was exposed to the saturated nitrobenzene vapor at room temperature. Figure 5 displays the time-dependent fluorescence quenching of n-TPE-AP-PDMS film to the saturated nitrobenzene vapor. The n-TPE-AP-PDMS film showed an emission peak at 532 nm. At the exposure time of 60 s, the fluorescence intensity of n-TPE-AP-PDMS film was quenched by 28 ± 2% (quenching efficiency, (*I*_0_ − *I*)/*I_0_* × 100%, where *I_0_* and *I* are luminescence intensity of n-TPE-AP-PDMS before and after exposure to nitrobenzene, respectively). The quenching efficiency reaches as high as 53 ± 2% at 600 s. These results indicate that n-TPE-AP-PDMS is a promising fluorescence sensor for nitrobenzene.

**Figure 6:**
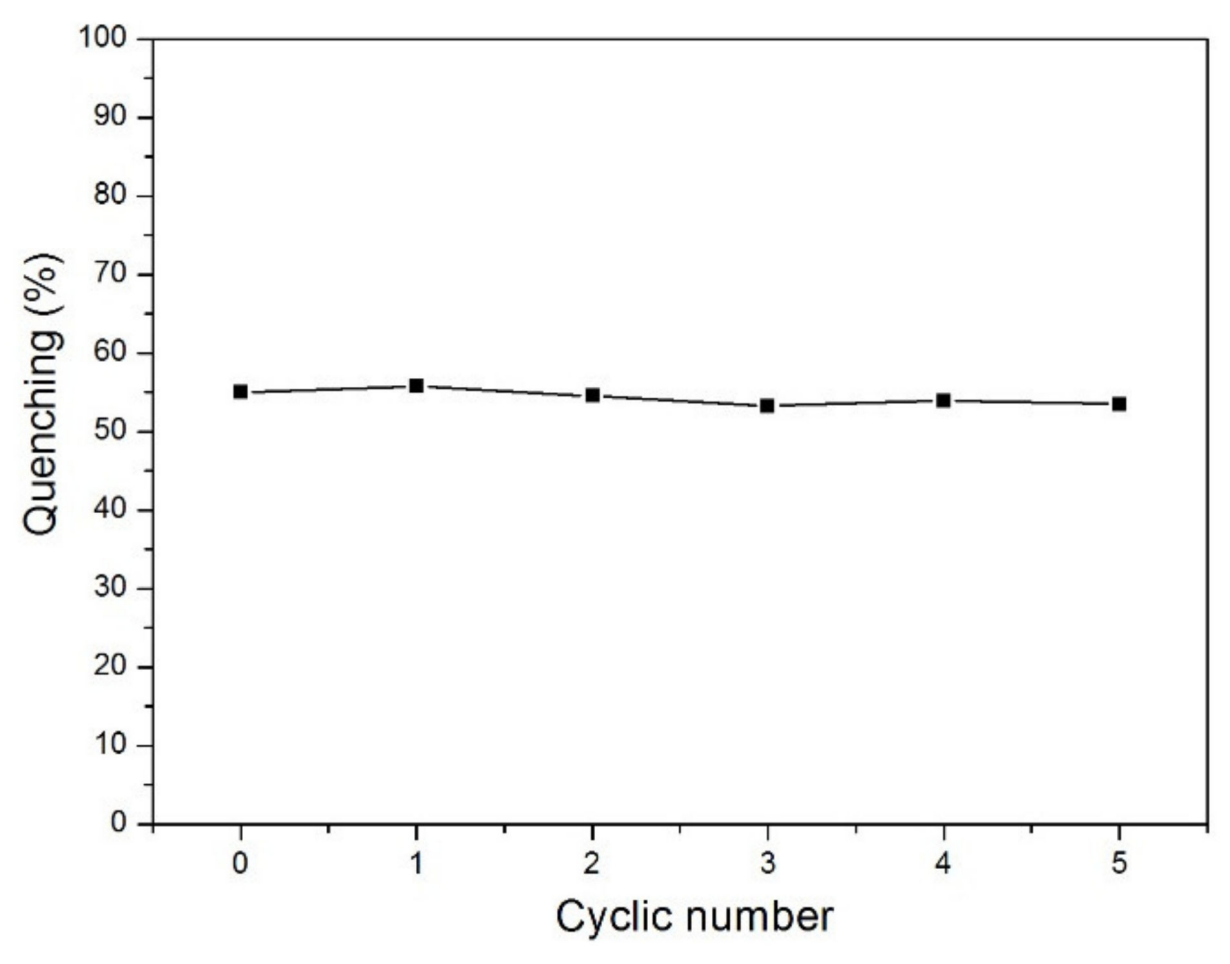
PL recovery cycles of n-TPE-AP-PDMS film in NB.

The reversibility of n-TPE-AP-PDMS film was further tested. The n-TPE-AP-PDMS film was exposed to saturated nitrobenzene vapors at room temperature for 600 s and the emission spectra were recorded. For fluorescence recovery, the film was dried at 50 °C for 3 h in a galvanothermal blast drying box; then, the emission spectra were measured again. As shown in Figure 6, after five cycles, the fluorescence quenching efficiency of n-TPE-AP-PDMS film for nitrobenzene remained almost the same. Meanwhile, the fluorescence quantum yields of n-TPE-AP-PDMS film with and without nitrobenzene were calculated to be 7.4% and 23.9%, respectively. Moreover, the n-TPE-AP-PDMS film has high selectivity for nitrobenzene compared to other common volatile solvents (Figure S5). This result from the reversibility analysis indicates that n-TPE-AP-PDMS film has great potential in practical applications for detecting nitrobenzene.

**Figure 7:**
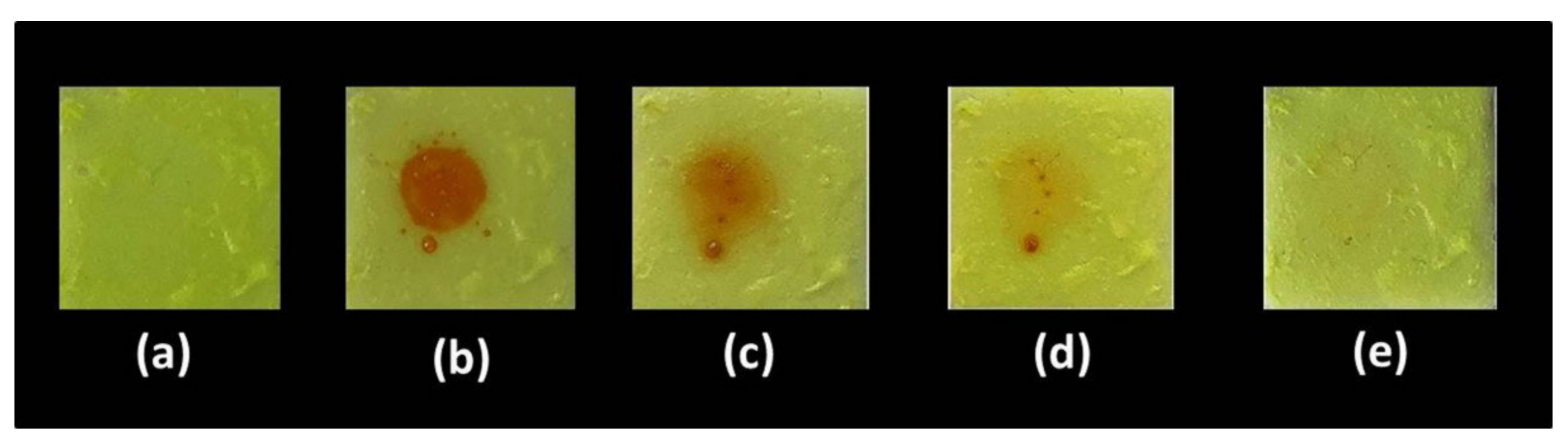
(**a**–**e**) Photographs of the fluorescence quenching and recovery of n-TPE-AP-PDMS film to NB liquid at different times in natural light.

We attempted to investigate the UV spectrum of nitrobenzene and the PL spectrum of n-TPE-AP-PDMS to account for this fluorescence quenching. From Figure S6, there is no discernible spectral overlap of the absorption of nitrobenzene and the emission of n-TPE-AP-PDMS films, indicating that the energy-transfer plays a negligible role in the fluorescence quenching. Therefore, the main quenching mechanism is charge-transfer between the excited state of n-TPE-AP-PDMS and the ground state of the nitrobenzene. Moreover, the response of n-TPE-AP-PDMS film in detecting nitrobenzene liquid can be visualized by the naked eye. Figure 7 shows the color changes in natural light after nitrobenzene solution was dropped on the n-TPE-AP-PDMS film. The n-TPE-AP-PDMS film was bright yellow in natural light (a). After adding a drop of nitrobenzene liquid on the film, the position of the nitrobenzene droplets immediately turned orange-red (b). Then, by using a blower to volatilize the nitrobenzene droplet, films (c) and (d), with a gradually diminishing orange–red color, could be obtained. Eventually, the n-TPE-AP-PDMS film with the orange–red color completely disappeared and bright yellow recovered (e). In addition, the n-TPE-AP-PDMS film that recovered to bright yellow can be used for nitrobenzene liquid detection again and showed good reversibility. The results proved that the n-TPE-AP-PDMS film had excellent response to nitrobenzene and great practical value.

## 4. Conclusions

In summary, we demonstrated the synthesis of an AIE silicone polymer (n-TPE-AP-PDMS) based on tetraphenylethylene via aza-Michael reaction. The introduction of polydimethylsiloxane to TPE showed no obvious effect on photophysical properties. n-TPE-AP-PDMS exhibited two opposite fluorescence emission behaviors in different systems: ACQ in tetrahydrofuran/water mixtures and AIE in tetrahydrofuran/hexane mixtures. This unusual transition from ACQ to AIE can be attributed to twisted intramolecular charge-transfer effect and flexible aminopropyl polydimethylsiloxane. In addition, n-TPE-AP-PDMS was used as a fluorescent probe for detecting nitrobenzene and showed high quenching efficiency. The n-TPE-AP-PDMS film showed high reversibility so that the quenching efficiency remains unchanged after five cycles. Moreover, the response of n-TPE-AP-PDMS film in detecting nitrobenzene liquid can be visualized by the naked eye. This work can provide a promising method to prepare high performance fluorescent probes.

## Data Availability

Not applicable.

## Supplementary Materials

The following are available online at https://www.mdpi.com/article/10.3390/polym13183046/s1, Figure S1: FT-IR spectra of n-TPE and n-TPE-AP-PDMS; Figure S2: SEM image of n-TPE-AP-PDMS; Figure S3: The absorption spectrum of NB and the PL spectrum of n-TPE- AP-PDMS.
