# Supplementary material for: Unexpected Fluorescence Emission Behaviors of Tetraphenylethylene-Functionalized Polysiloxane and Highly Reversible Sensor for Nitrobenzene
Source: Polymers (Basel). 2021 Sep 9;13(18):3046. doi: 10.3390/polym13183046 (PMC8470815; doi:10.3390/polym13183046)
Supplement: Supplementary file 1 [file polymers-13-03046-s001.zip › polymers-1326292-supplementary.pdf]

Supplementary Information

Unexpected Fluorescence Emission Behaviors of Tetraphenylethylene Functionalized Polysiloxane and Highly Reversible Sensor for Nitrobenzene

Lianfeng Wu, Qin Jiang, Haifeng Lu*, and Shengyu Feng*

Key Laboratory of Special Functional Aggregated Materials (Shandong University), Ministry of Education, School of Chemistry and Chemical Engineering, Shandong University, Jinan, 250100, P.R. China

Corresponding authors: lhf@sdu.edu.cn (H.L.)
fsy@sdu.edu.cn (S.F.)

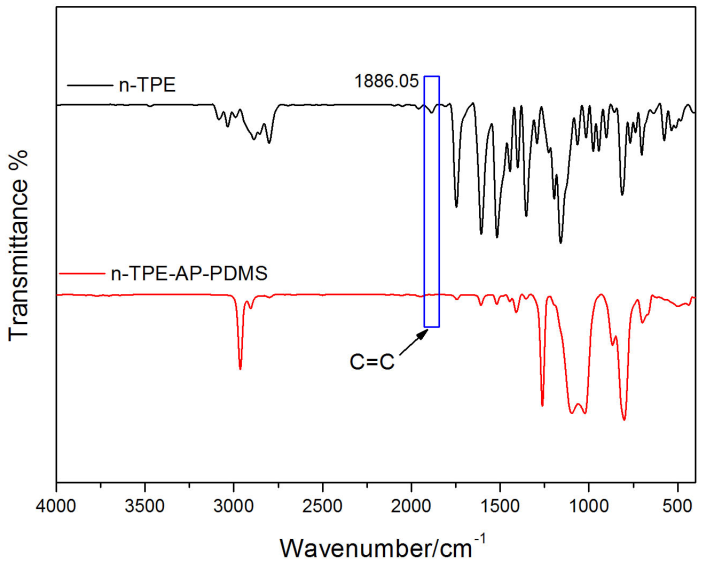

Figure S1. FTIR spectra of n-TPE and n-TPE-AP-PDMS.

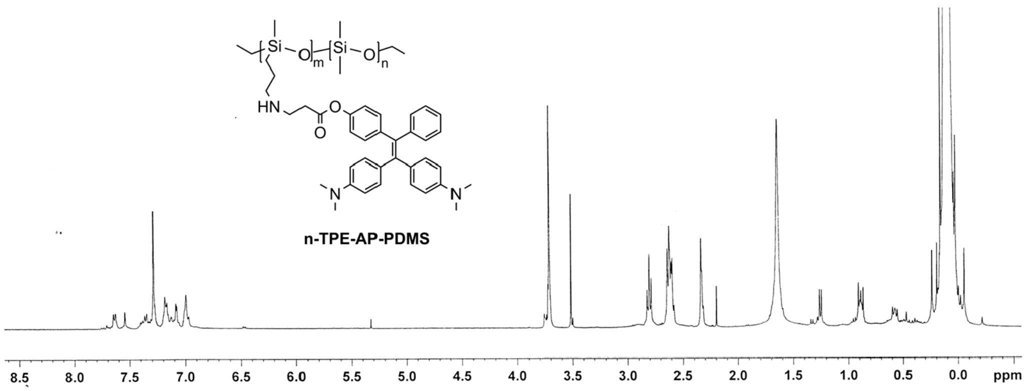

Figure S2. The ^{1}H NMR spectrum of n-TPE-AP-PDMS

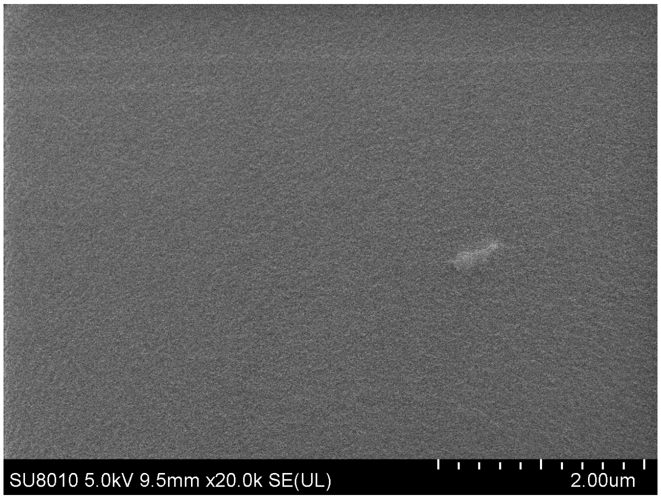

Figure S3. SEM image of n-TPE-AP-PDMS.

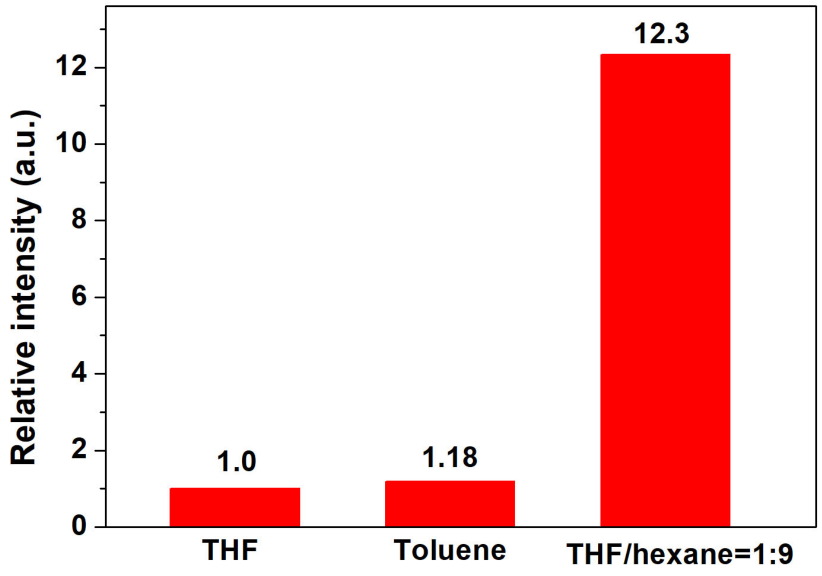

Figure S4. The relative PL intensity of n-TPE-AP-PDMS in different solvents (concentration: 10 μM)

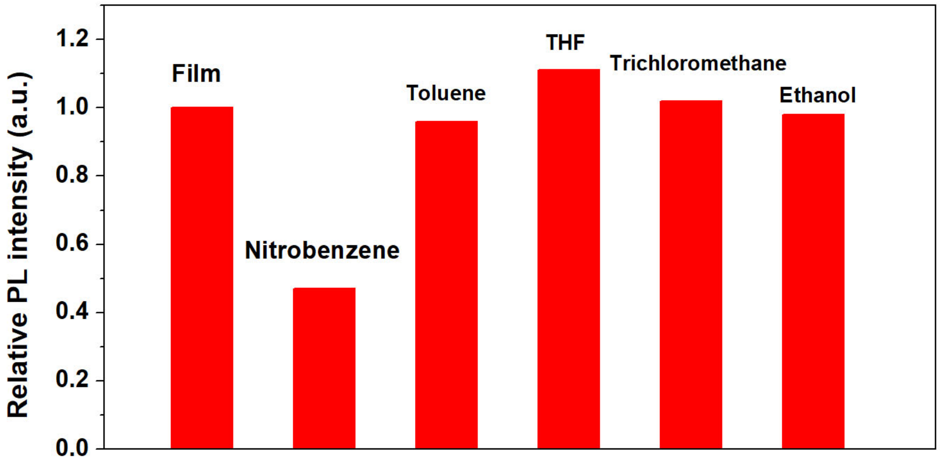

Figure S5. The PL intensity of n-TPE-AP-PDMS with nitrobenzene compared to other common volatile solvents

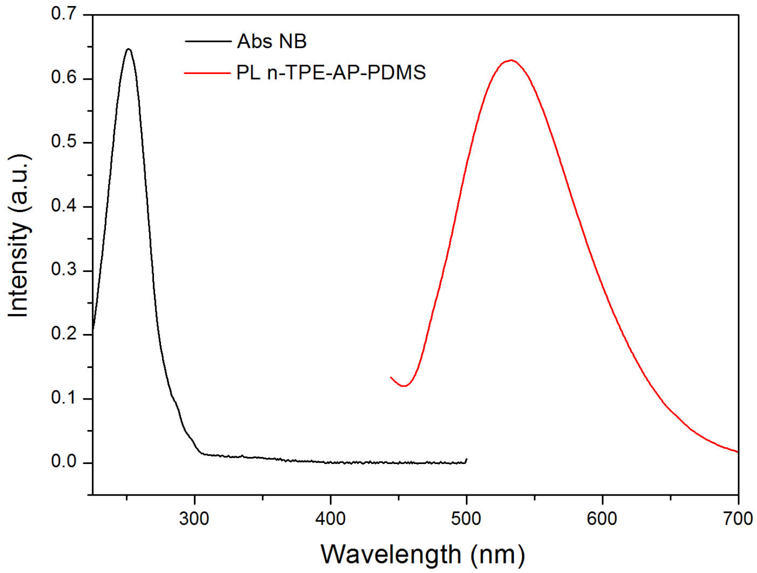

Figure S6. The absorption spectrum of NB and the PL spectrum of n-TPE- AP-PDMS.
